# Comparative proteomic analysis of glomerular proteins in IgA nephropathy and IgA vasculitis with nephritis

**DOI:** 10.1186/s12014-023-09409-w

**Published:** 2023-05-13

**Authors:** Hajime Kaga, Hirotoshi Matsumura, Ayano Saito, Masaya Saito, Fumito Abe, Takehiro Suzuki, Naoshi Dohmae, Masafumi Odaka, Atsushi Komatsuda, Hideki Wakui, Naoto Takahashi

**Affiliations:** 1 Department of Hematology, Nephrology, and Rheumatology, Akita University Graduate School of Medicine, Akita, Japan; 2 Department of Life Science, Graduate School of Engineering Science, Akita University, Akita, Japan; 3 Biomolecular Characterization Unit, RIKEN Center for Sustainable Resource Science, Wako, Japan; 4 Department of Internal Medicine, Ogachi Central Hospital, Yuzawa, Japan; 5 Emeritus Professor, Akita University, Akita, Japan

**Keywords:** Comparative proteomic analysis, Glomerular proteins, IgA nephropathy, IgA vasculitis with nephritis, Mass spectrometry

## Abstract

**Background:**

IgA nephropathy (IgAN) and IgA vasculitis with nephritis (IgAVN) are related glomerular diseases characterized by marked similarities in immunological and histological findings. We herein performed a comparative proteomic analysis of glomerular proteins in IgAN and IgAVN.

**Methods:**

We used renal biopsy specimens from 6 IgAN patients without nephrotic syndrome (NS) (IgAN-I subgroup), 6 IgAN patients with NS (IgAN-II subgroup), 6 IgAVN patients with 0–8.0% of glomeruli with crescent formation (IgAVN-I subgroup), 6 IgAVN patients with 21.2–44.8% of glomeruli with crescent formation (IgAVN-II subgroup), 9 IgAVN patients without NS (IgAVN-III subgroup), 3 IgAVN patients with NS (IgAN-IV subgroup), and 5 control cases. Proteins were extracted from laser microdissected glomeruli and analyzed using mass spectrometry. The relative abundance of proteins was compared between groups. An immunohistochemical validation study was also performed.

**Results:**

More than 850 proteins with high confidence were identified. A principal component analysis revealed a clear separation between IgAN and IgAVN patients and control cases. In further analyses, 546 proteins that were matched with ≥ 2 peptides were selected. The levels of immunoglobulins (IgA, IgG, and IgM), complements (C3, C4A, C5, and C9), complement factor H-related proteins (CFHR) 1 and 5, vitronectin, fibrinogen chains, and transforming growth factor-β inducible gene-h3 were higher (> 2.6 fold) in the IgAN and IgAVN subgroups than in the control group, whereas hornerin levels were lower (< 0.3 fold). Furthermore, C9 and CFHR1 levels were significantly higher in the IgAN group than in the IgAVN group. The abundance of some podocyte-associated proteins and glomerular basement membrane (GBM) proteins was significantly less in the IgAN-II subgroup than in the IgAN-I subgroup as well as in the IgAVN-IV subgroup than in the IgAVN-III subgroup. Among the IgAN and IgAVN subgroups, talin 1 was not detected in the IgAN-II subgroup. This result was supported by immunohistochemical findings.

**Conclusions:**

The present results suggest shared molecular mechanisms for glomerular injury in IgAN and IgAVN, except for enhanced glomerular complement activation in IgAN. Differences in the protein abundance of podocyte-associated and GBM proteins between IgAN and IgAVN patients with and without NS may be associated with the severity of proteinuria.

**Supplementary Information:**

The online version contains supplementary material available at 10.1186/s12014-023-09409-w.

## Background

Immunoglobulin (Ig) A nephropathy (IgAN) is the most common type of primary glomerulonephritis worldwide and a leading cause of chronic kidney disease progressing to renal failure in adults [1]. It is histologically characterized by dominant or co-dominant IgA deposits, typically with complement C3 and variable amounts of IgG and IgM, in glomerular mesangial areas [1].

Recent advances in our understanding of the pathogenesis of IgAN have led to the proposal of a four-hit hypothesis [1]. Hit 1 is the increased production of circulating galactose-deficient IgA1 (Gd-IgA1), which is considered to originate from cells in mucosal tissues. Hit 2 is the production of circulating IgG or IgA autoantibodies specific for Gd-IgA1. Hit 3 is the formation of circulating pathogenetic Gd-IgA1–containing immune complexes. Hit 4 is the glomerular deposition of these immune complexes, resulting in the activation and proliferation of mesangial cells, the release of inflammatory cytokines, and renal injury. The activation of the complement system induces the additional secretion of inflammatory mediators and matrix proteins by mesangial cells [1]. The consequence of this is an increase in glomerular permeability mainly due to podocyte injury, leading to persistent proteinuria and renal dysfunction during an asymptomatic disease course (podocytopathy) [2]. Approximately 5% of IgAN patients develop nephrotic syndrome (NS) with advanced glomerulosclerosis [1].

IgA vasculitis (IgAV), formerly known as Henoch-Schönlein purpura, is a systemic disease affecting the skin, joints, gastrointestinal tract, and kidneys. When the kidneys are affected, the disease is termed IgAV with nephritis (IgAVN) [1, 3]. Renal involvement is more common among children than adults, but is more severe in adults. IgAVN is characterized by proliferative glomerulonephritis with the predominant deposition of IgA, which is indistinguishable from the histological findings of IgAN [1, 3]. In addition, IgAN and IgAVN have a shared feature involving a Gd-IgA1–oriented pathogenesis [1, 3, 4]. Therefore, the 2 diseases are considered to be closely related [5]. We previously suggested that the up-regulated expression of Toll-like receptors in peripheral blood mononuclear cells, key components of innate immune responses against diverse pathogens, plays important roles in the initiation of the pathogenetic process in IgAN and IgAVN [6].

Proteomic studies will advance our understanding of the pathophysiology of glomerular diseases. We recently performed a comparative proteomic analysis of whole kidney extracts from IgAN model (HIGA) mice and control mice [7]. The levels of various proteins that are known to be associated with kidney diseases, including IgAN, in humans and/or animal models of kidney diseases differed in HIGA mice. We also performed a comparative proteomic analysis of glomerular proteins extracted from laser microdissected glomeruli in patients with primary membranous nephropathy (MN) and drug-induced secondary MN and control cases [8]. The findings obtained suggested common and different pathogenetic mechanisms between primary MN and drug-induced secondary MN.

Urine and/or serum samples were mainly analyzed in previous proteomic approaches to IgAN and IgAVN [9, 10]. Recent studies examined the profiles of Igs, complements, complement-regulating proteins, glomerular basement membrane (GBM) proteins, and extracellular matrix (ECM)-associated proteins in IgAN using laser microdissected glomeruli from formalin-fixed paraffin-embedded tissue sections [11–13]. Regarding the relationship between IgAN and IgAVN, Fang et al. [14] recently reported the findings of a urinary proteomic analysis of pediatric patients and suggested that several common pathways play important roles in the progression of IgAN and IgAVN.

In the present study, we performed laser microdissection and a comparative proteomic analysis of glomerular proteins from patients with IgAN and adult-onset IgAVN and control cases. We then characterized the profiles of several protein groups based on structural and functional characteristics, such as Igs, complements, complement-regulating proteins, podocyte-associated proteins, GBM proteins, and ECM-associated proteins, and found similar protein profiles between IgAN and IgAVN. The results obtained also showed that the abundance of some proteins significantly differed between IgAN and IgAVN patients and between IgAN and IgAVN patients with and without NS.

## Methods

### Patients

We enrolled 12 patients with IgAN, 12 with IgAVN, and 5 healthy transplantation donors (time 0 transplant biopsies) as controls in the proteomics study. These patients and donors underwent renal biopsy between February 2011 and May 2021 at Akita University Hospital and its affiliated hospitals. All patients and donors were Japanese. IgAN was diagnosed based on renal biopsy findings [1], and none of the patients presented with clinical findings suggestive of the causes of secondary IgAN [1]. IgAVN was diagnosed according to the EULAR/PRINTO/PRES criteria [15]: purpura with a lower limb predominance, urinary abnormalities, and proliferative glomerulonephritis with predominant IgA deposits. Skin biopsy was performed on 2 patients, and biopsy findings were compatible with IgAVN.

According to the degree of proteinuria (one of the prognostic factors for IgAN [1]), IgAN patients were divided into 2 subgroups: 6 patients without NS (IgAN-I subgroup) and 6 with NS (IgAN-II subgroup). The degree of glomerular crescent formation is one of the prognostic factors for IgAVN [3]. All IgAVN patients showed grade II mesangial proliferation or grade III mesangial proliferation with crescents (< 50% of glomeruli) [3], and the crescent formation rates in 12 patients were 0, 2.5, 2.7, 4.8, 6.7, 8.0, 21.2, 25.7, 27.9, 31.3, 33.3, and 44.8%. Accordingly, IgAVN patients were divided into 2 subgroups: 6 patients with 0–8.0% of glomeruli with crescent formation (IgAVN-I subgroup) and 6 with 21.2–44.8% of glomeruli with crescent formation (IgAVN-II subgroup). IgAVN patients were also divided into 2 subgroups according to the degree of proteinuria: 9 patients without NS (IgAVN-III subgroup) and 3 with NS (IgAVN-IV subgroup).

In the immunohistochemical validation study, we enrolled an additional 6 Japanese patients with IgAN (3 without NS and 3 with NS). These patients underwent renal biopsy between August 2018 and June 2021. IgAN was diagnosed based on the findings obtained, as described above.

### Clinicopathological analysis

The clinical data of patients and transplantation donors were collected from medical records as previously described [8]. Hypertension was defined as systolic blood pressure ≥ 140 mmHg, diastolic blood pressure ≥ 90 mmHg, or the use of antihypertensive medications at the time of biopsy. NS was defined as urinary protein ≥ 3.5 g/day or g/g creatinine (Cr) and hypoalbuminemia (serum albumin ≤ 3.0 g/dL).

Renal biopsy specimens were processed using standard techniques as previously described [8]. The histological severities of IgAN and IgAVN were assessed according to the Oxford classification of IgAN [1] and the International Study of Kidney Disease in Children (ISKDC) classification of IgAVN [3], respectively. The Oxford classification includes the following 5 key pathological features [1]: mesangial hypercellularity (M), endocapillary hypercellularity (E), segmental glomerulosclerosis (S), tubular atrophy and interstitial fibrosis (T), and cellular or fibrocellular crescents (C).

Cryostat sections for immunofluorescence microscopy were stained with fluorescein isothiocyanate (FITC)-conjugated rabbit polyclonal antibodies against human IgG, IgA, IgM, κ, λ, C3, and C1q (Dako Cytomation, Glostrup, Denmark) as previously described [8]. Further studies to identify IgA subclasses were performed using mouse monoclonal antibodies against human IgA1 and IgA2 (Nordic-MUbio, Susteren, the Netherlands) and FITC-conjugated anti-mouse Igs (Agilent Technologies, Singapore, Malaysia).

An immunohistochemical study on biopsy specimens from 6 IgAN patients with and without NS was also performed. Sections were processed with a rabbit polyclonal antibody to human talin 1 (ab71333, Abcam, Cambridge, UK). Sections were then stained using N-Histofine® Simple Stain MAX PO (MULTI) (Nichirei Biosciences, Tokyo, Japan) and the ImmPACT™ DAB substrate kit (Vector Laboratories, Burlingame, CA, USA). Counterstaining was conducted with hematoxylin. Glomerular talin 1 staining-positive area ratios (T1S-PARs) were semi-quantitatively evaluated on 3 glomeruli in each patient using the open-access software ImageJ/Fiji [16].

### Laser microdissection and a proteomic analysis

Laser microdissection and a comparative proteomic analysis of glomerular proteins were performed as previously described [8]. In brief, non-sclerotic glomerular tufts were microdissected from formalin-fixed paraffin-embedded renal sections, excluding crescentic lesions, as reported by Paunas et al. [12]. Microdissected glomeruli were pooled in microcentrifuge tubes to reach approximately 760,000–4,000,000 µm^2^ per case (the total number of glomeruli was 53–187). Dissected glomeruli were treated with approximately 100–200 µL of TE Buffer (Promega Corporation, Madison, WI, USA) containing 0.002% hexadecyldimethyl(3-sulfopropyl) ammonium hydroxide inner salt (Tokyo Medical Industry, Tokyo, Japan). Glomerular proteins were extracted in microcentrifuge tubes by sonication.

Nano-liquid chromatography-tandem mass spectrometry (nLC-MS/MS) was performed as previously described [8]. In brief, extracted glomerular proteins were subjected to sodium dodecyl sulfate–polyacrylamide gel electrophoresis. Electrophoresis was stopped when the dye front migrated from the stacking gel to the separation gel. Protein bands were excised and subjected to in-gel trypsin digestion. Tryptic digests were separated on Easy-nLC 1200 (Thermo Fisher Scientific, Waltham, MA, USA) using a C18 analytical column (NTCC-360/75-3-155, Nikkyo Technos, Tokyo, Japan). Separated peptides were then analyzed on a Q-Exactive HF-X mass spectrometer (Thermo Fisher Scientific) coupled with Easy-nLC 1200 (Thermo Fisher Scientific).

### Data analysis

Data analyses were performed as previously described [8]. The identification and quantification of peptides and proteins were performed using a commonly used label-free quantification method [17]. In brief, data obtained in nLC-MS/MS experiments were analyzed using Proteome Discoverer 2.4 (Thermo Fisher Scientific; https://assets.thermofisher.com/TFS-Assets/CMD/manuals/Man-XCALI-97808-Proteome-Discoverer-User-ManXCALI97808-EN.pdf) and Mascot Server 2.7 (Matrix Science; https://www.matrixscience.com/server.html). The National Center for Biotechnology Information non-redundant database (NCBInr) is a predefined database for protein identification. When an NCBInr search did not provide a unique protein name, a Blast search (https://blast.ncbi.nlm.nih.gov/Blast.cgi) was performed. The reproducibility of the data obtained for samples from each group was confirmed through a principal component analysis. Fold-change values for protein abundance were compared between each subgroup of IgAN and IgAVN and the control group (expressed as IgAN-I/C, IgAN-II/C, IgAVN-I/C, IgAVN-II/C, IgAVN-III/C, and IgAVN-IV/C ratios). If the denominator of a fraction was zero or almost zero, the relative protein abundance ratio was defined as 100. If the numerator of a fraction was zero or almost zero, the relative protein abundance ratio was defined as 0.01.

### Statistical analysis

The normalized protein abundance of Igs, complements, complement-regulating proteins, podocyte-associated proteins, GBM proteins, and ECM-associated proteins were compared between groups. Glomerular T1S-PARs were compared between IgAN patients without and with NS. Data were expressed as mean ± SD. Based on the results of Levene’s test for the equality of variance, the Student’s *t*-test or Welch’s *t*-test was used. *P*-values of < 0.05 were considered to be significant. Relative differences in protein abundance are given as fold changes between groups.

## Results

### Clinicopathological characteristics of patients with IgAN and IgAVN and transplantation donors

The clinical features of patients with IgAN and IgAVN and transplantation donors are shown in Table 1. Patients with IgAN and IgAVN were adults, except for 1 IgAN patient. The median ages of patients in the IgAN-I, IgAN-II, IgAVN-I, and IgAVN-II subgroups, and healthy donors were 42, 67, 71, 60, and 53 years, respectively. In the IgAN-I subgroup, all patients presented with chance proteinuria and/or hematuria, and 2 had hypertension. In the IgAN-II subgroup, all patients presented with progressive renal dysfunction and/or edema, and 4 had hypertension. In the IgAVN group, all patients presented with urinary abnormalities with progressive renal dysfunction and/or edema after the onset of purpura, and 5 had hypertension. Commonly used antihypertensive drugs were renin*-*angiotensin system inhibitors and calcium channel blockers. In the IgAVN-II subgroup, 1 patient presented with arthralgia, while another had abdominal pain.

**Table 1:** Clinical characteristics of patients with IgAN and IgAVN included in the present study and control subjects

	IgAN-I	IgAN-II	IgAVN-I	IgAVN-II	Controls
Number of patients and controls	6	6	6	6	5
Median age (years) (range)	42 (16‒71)	67 (22‒76)	71 (36‒78)	60 (34‒74)	53 (45‒77)
Male:female	3:3	2:4	2:4	2:4	1:4
Chance proteinuria and/or hematuria, *n*	6	0			
Urinary abnormalities after the onset of purpura, *n*			6	6	
Progressive renal dysfunction, *n*	1	3	0	1	
Edema, *n*	0	5	0	2	
Hypertension, *n*	2	4	1	4	
RAS-I/CCB/SARA/AB/BB/DU, *n*	2/1/0/0/0/0	1/3/1/1/0/0	1/1/0/0/0/0	3/2/0/1/1/2	
Purpura, *n*			6	6	
Arthralgia, *n*			0	1	
Abdominal pain, *n*			0	1	
Median proteinuria (g/day or g/gCr) at biopsy (range)	0.4 (0.1–2.8)	5.9 (4.1‒10.0)	3.6 (0.2‒7.9)	2.0 (0.5‒6.4)	0.7 (0.1‒0.9)
Median serum albumin (g/dL) (range)	4.0 (3.5‒4.7)	2.5 (2.2‒2.8)	3.5 (1.8‒4.3)	3.6 (2.5‒3.9)	4.3 (3.5‒4.5)
Nephrotic syndrome, *n*	0	6	1	2	
Median serum Cr (mg/dL) (range)	0.89 (0.50‒1.85)	1.13 (0.62‒2.07)	0.79 (0.48‒1.18)	0.76 (0.59‒2.10)	0.67 (0.60‒0.90)
Median eGFR (mL/min/1.73 m^2^) (range)	77.8 (32.6‒109.3)	55.6 (22.4‒73.6)	66.0 (34.5‒98.4)	59.2 (25.1‒92.8)	72.1 (66.9‒81.6)
Median serum IgA (mg/dL) (range)	439 (213‒921)	322 (189‒528)	305 (254‒410)	330 (214‒382)	

AB: α1 blocker; BB: β blocker; CCB: calcium channel blocker; Cr: creatinine; DU: diuretics; eGFR: estimated glomerular filtration rate; IgAN: IgA nephropathy; IgAVN: IgA vasculitis with nephritis; RAS-I: renin-angiotensin system inhibitor; SARA: selective aldosterone receptor antagonist

The median levels of proteinuria were ≥ 3.5 g/day or g/gCr in the IgAN-II and IgAVN-I subgroups. The median level of serum albumin was ≤ 3.0 g/dL in the IgAN-II subgroup. All patients in the IgAN-II subgroup developed NS. In the IgAVN group, 3 patients developed NS. The median levels of the estimated glomerular filtration rate were < 60 mL/min/1.73 m^2^ in the IgAN-II and IgAVN-II subgroups. The median levels of serum IgA were elevated in the IgAN and IgAVN subgroups.

The pathological features of our patients with IgAN and IgAVN are shown in Table 2. The median times from clinical presentation to biopsy in the IgAN-I, IgAN-II, IgAVN-I, and IgAVN-II subgroups were 20, 5, 2, and 4 months, respectively. Clinical presentations that prompted biopsy were chance proteinuria and/or hematuria in the IgAN-I subgroup, progressive renal dysfunction and/or edema in the IgAN-II subgroup, and urinary abnormalities after the onset of purpura in the IgAVN group. In the IgAVN group, 5 patients experienced the relapse of purpura following spontaneous improvements before biopsy.

**Table 2:** Pathological characteristics of patients with IgAN and IgAVN included in the present study and control subjects

	IgAN-I	IgAN-II	IgAVN-I	IgAVN-II
Median time from presentation to biopsy (months) (range)	20 (6–40)	5 (0.5-9)	2 (0.5–12) #	4 (0.5–15) #
Median % of global sclerotic glomeruli (range)	9.7 (0-44.4)	35.3 (2.9–47.1)	7.8 (0–16.0)	7.5 (2.5–23.8)
Oxford classification, *n*				
M score	1 M0, 5 M1	2 M0, 4 M1		
E score	3 E0, 3 E1	5 E0, 1 E1		
S score	3 S0, 3 S1	3 S0, 3 S1		
T score	4 T0, 2 T1	3 T0, 1 T1, 2 T2		
C score	2 C0, 4 C1	1 C0, 4 C1, 1 C2		
ISKDC classification grade, *n*			1 II, 2 IIIa, 3 IIIb	1 IIIa, 5 IIIb
% of glomeruli with crescent formation			0, 2.5, 2.7, 4.8, 6.7, 8.0	21.2, 25.7, 27.9, 31,3, 33.3, 44.8
Glomerular deposition (IF intensities), *n*				
IgA	5 (2+), 1 (3+)	2 (+), 4 (2+)	2 (+), 4 (2+)	1 (+), 5 (2+)
IgA1	6 (+)	5 (+), 1 (2+)	5 (+), 1 (2+)	1 (±), 4 (+), 1 (2+)
IgA2	6 (‒)	6 (‒)	6 (‒)	5 (‒), 1 (±)
IgG	3 (‒), 1 (±), 2 (+)	3 (‒), 1 (±), 2 (+)	3 (‒), 1 (±), 2 (+)	4 (‒), 1 (±), 1 (+)
IgM	1 (‒), 3 (±), 2 (+)	2 (‒), 2 (±), 2 (+)	4 (‒), 1 (±), 1 (+)	4 (‒), 1 (±), 1 (+)
κ	4 (+), 2 (2+)	2 (±), 2 (+), 2 (2+)	2 (±), 2 (+), 2 (2+)	2 (‒), 3 (+), 1 (2+)
λ	4 (+), 2 (2+)	1 (±), 2 (+), 3 (2+)	6 (+)	6 (+)
C3	4 (+), 2 (2+)	1 (‒), 1 (±), 1 (+), 3 (2+)	1 (‒), 3 (+), 2 (2+)	1 (‒), 1 (±), 2 (+), 2 (2+)
C1q	5 (‒), 1 (±)	6 (‒)	6 (‒)	6 (‒)

C: cellular or fibrocellular crescents; E: endocapillary hypercellularity; IF: immunofluorescence; IgAN: IgA nephropathy; IgAVN: IgA vasculitis with nephritis; ISKDC: International Study of Kidney Disease in Children; M: mesangial hypercellularity; S: segmental glomerulosclerosis; T: tubular atrophy and interstitial fibrosis

# Purpura relapsed in 2 patients in the I subgroup and 3 in the II subgroup following spontaneous remission. The durations from the first episode of purpura to biopsy in these patients are described.

The median percentages of global sclerotic glomeruli in the IgAN-I, IgAN-II, IgAVN-I, and IgAVN-II subgroups were 9.7, 35.3, 7.8, and 7.5%, respectively. M and S scores in the Oxford classification [1] were similar between the IgAN-I and -II subgroups. E1 lesions were observed in 3 patients in the IgAN-I subgroup and 1 in the IgAN-II subgroup. In the IgAN-II subgroup, T2 and C2 lesions were detected in 2 patients and 1 patient, respectively. All IgAVN patients showed mesangial proliferation (grade II) or focal or diffuse mesangial proliferation with < 50% of glomeruli with crescent formation (grade IIIa or IIIb) in the ISKDC system [3]. The percentages of glomeruli with crescent formation in the IgAVN-I and -II subgroups were 0–8.0 and 21.2–44.8%, respectively. On immunofluorescence microscopy, glomerular IgA deposition was noted in all patients with IgAN and IgAVN, and the co-deposition of IgG and IgM was observed in some patients. Positive staining for IgA1 and negative staining for IgA2 were detected in most patients with IgAN and IgAVN. Positive staining for Ig κ and λ light chains and complement C3 and negative staining for complement C1q were observed in most patients with IgAN and IgAVN.

The clinicopathological features of IgAN patients without and with NS included in the immunohistochemical study are shown in Supplementary Tables S1 and S2 (Additional files 1 and 2).

### Comparative proteomic analysis

**Fig. 1:**
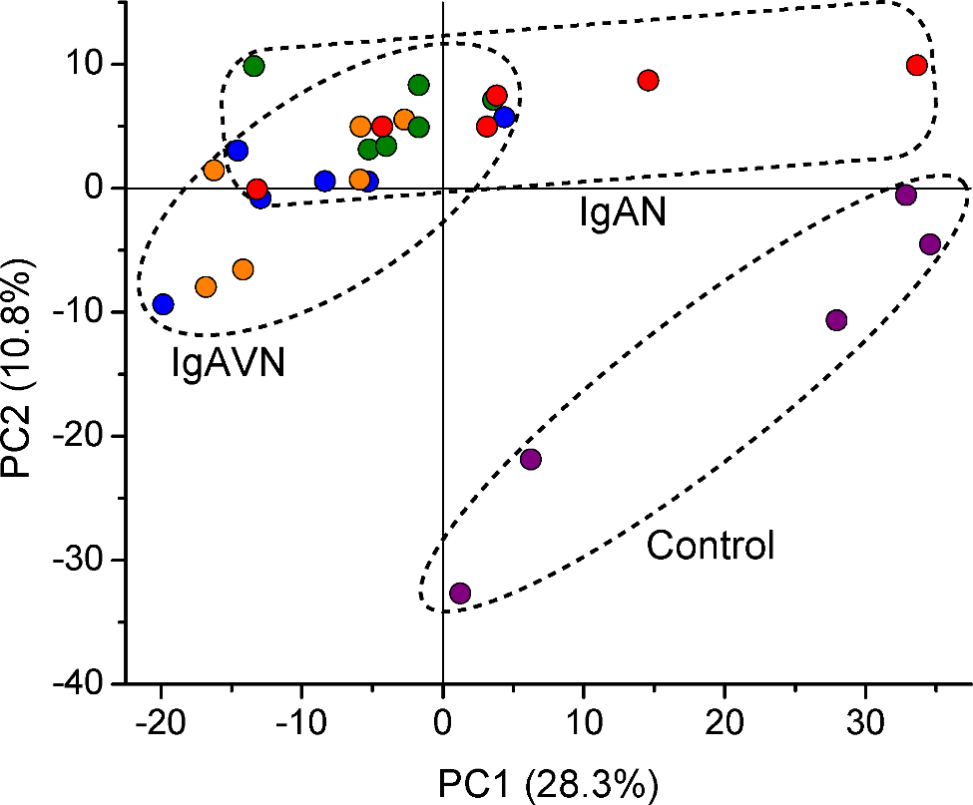
Principal component analysis. Data obtained from 6 IgAN patients without NS (IgAN-I), 6 IgAN patients with NS (IgAN-II), 6 IgAVN patients with 0–8.0% of glomeruli with crescent formation (IgAVN-I), 6 IgAVN patients with 21.2–44.8% of glomeruli with crescent formation (IgAVN-II), and 5 control cases (time 0 transplant biopsies) are shown. Data from the IgAN-I, IgAN-II, IgAVN-I, and IgAVN-II subgroups and the control group are represented by green, red, orange, blue, and purple circles, respectively. Variance is given as a percentage for both the first and second principal components (PC1 and PC2).

In our proteomic analysis of glomerular proteins extracted from the laser microdissected glomeruli of the enrolled subjects (Additional file 3: Table S3), a principal component analysis revealed a clear separation between the IgAN and IgAVN groups and the control group and an overlapping distribution between the IgAN and IgAVN groups (Fig. 1). Supplementary Table S4 (Additional file 4) summarizes the peptides detected and used to compare protein abundance.

We identified 859 proteins with high confidence (experimental *Q* < 0.01), and further selected 546 proteins that were matched with ≥ 2 peptides. Among them, 300 proteins were commonly detected in the IgAN and IgAVN groups and the control group, and 21 proteins were identified in all patients and controls. Proteins that increased or decreased in the IgAN and IgAVN subgroups with medium or high confidence (adjusted *P* < 0.05 or < 0.01) are shown in Tables 3, 4, 5, 6, and 7.

**Table 3:** List of immunoglobulins

Accession ID NCBInr DB	Accession ID GenBank or PDB DB	Protein name	Sequence coverage (%)	Peptide match (*n*)	IgAN-I (*n* = 6) / Control (*n* = 5) ratio (Ratio variability [%])	IgAN-II (*n* = 6) / Control (*n* = 5) ratio (Ratio variability [%])	IgAVN-I (*n* = 6) / Control (*n* = 5) ratio (Ratio variability [%])	IgAVN-II (*n* = 6) / Control (*n* = 5) ratio (Ratio variability [%])
34527290	AK130476.1	Ig α1 HC VC region	20	9	100 **	100 **	100 **	100 **
34527233	BAC85349.1	Ig α1 HC VC region	20	9	1.67 (106.05)	2.02 (96.64)	1.23 (98.81)	1.87 (84.43)
87783		Ig α2 HC C region	30	6	100 **	100 **	100 **	100 **
34527698	AK130813.1	Ig γ1 HC VC region	24	9	100 **	100 **	100 **	100 **
16553682	AK057754.1	Ig γ1 HC VC region	26	10	100 **	100 **	100 **	100 **
304562031		Ig γ2 HC V region	19	2	100 **	100 **	100 **	
10334541	AJ390237.1	Ig γ3 HC C region	21	6	0.52 (4.14)	1.16 (47.59)	0.60 (57.33)	1.14 (44.04)
953267710		Ig γ4 HC C region	16	5	100 **	100 **	100 **	
304563964		Ig γ4 HC V region	24	3		100 **	100 **	100 **
15825712	1HEZ_B	Ig µ HC VC region	13	2	100 **	100 **	100 **	100 **
33451	X17115.1	Ig µ HC C region	12	6	5.69 (60.78)	4.23 (45.37)	4.72 (56.46)	4.58 (49.63)
21669411		Ig κ LC VJ region	31	6	100 **	100 **	100 **	100 **
21669561		Ig λ LC VJ region	27	5	100 **	100 **	100 **	100 **
114319027		Ig joining chain	15	3	2.32 (71.68)	3.50 (45.89)	4.23 (16.85)	2.54 (34.33)

C: constant; DB: database; HC: heavy chain; Ig: immunoglobulin; IgAN: IgA nephropathy; IgAVN: IgA vasculitis with nephritis; LC: light chain; NCBInr: National Center for Biotechnology information non-redundant; PDB: Protein Data Bank; V: variable; VC: variable and constant; VJ: variable and joining

**P < 0.01

**Table 4:** List of complements and complement-regulating proteins

Accession ID NCBInr DB	Accession ID GenBank DB	Protein name	Sequence coverage (%)	Peptide Match (*n*)	IgAN-I (*n* = 6) / Control (*n* = 5) ratio (Ratio variability [%])	IgAN-II (*n* = 6) / Control (*n* = 5) ratio (Ratio variability [%])	IgAVN-I (*n* = 6) / Control (*n* = 5) ratio (Ratio variability [%])	IgAVN-II (*n* = 6) / Control (*n* = 5) ratio (Ratio variability [%])
115298678		C3	17	25	6.75 * (114.91)	5.35 * (107.18)	4.86 (93.25)	2.64 (92.76)
443671		C4A	4	4	4.51 (65.96)	4.72 (89.78)	3.12 (61.45)	4.00 (72.75)
953514538		C5	2	2	100 **	100 **	100 **	100 **
109731764		C8γ	13	2	2.00 (46.48)	2.19 (46.82)	0.65 (3.71)	1.08 (16.90)
119576392		C9	14	8	16.72 ** (98.31)	9.78 ** (113.13)	7.45 * (89.20)	4.24 (88.87)
183763		CFHR1	32	8	9.66 * (78.53)	5.58 * (111.92)	3.00 (107.85)	3.25 (84.79)
767910533		CFHR5	4	2	100 **	100 **	100 **	100 **
13477169		Vitronectin	15	7	3.82 (102.41)	3.75 (110.86)	2.84 (105.66)	3.03 (109.83)
578815184		Clusterin	20	13	2.48 (87.08)	2.41 (109.01)	1.81 (84.97)	1.57 (77.56)
4502503		C4BPA	4	2	1.71 (113.99)	1.89 (83.17)	0.80 (57.79)	0.63 (75.75)
809019	X05309.1	CR1	3	3	0.53 (115.68)	0.27 (123.83)	0.57 (86.45)	0.50 (106.89)

C4BPA: C4b-binding protein α chain; CFHR: complement factor H-related protein; CR1: complement receptor 1; DB: database; IgAN: IgA nephropathy; IgAVN: IgA vasculitis with nephritis; NCBInr: National Center for Biotechnology information non-redundant

*P < 0.05, **P < 0.01

**Table 5:** List of podocyte-associated proteins

Accession ID NCBInr DB	Accession ID GenBank DB	Protein name	Sequence coverage (%)	Peptide match (*n*)	IgAN-I (*n* = 6) / Control (*n* = 5) ratio (Ratio variability [%])	IgAN-II (*n* = 6) / Control (*n* = 5) ratio (Ratio variability [%])	IgAVN-I (*n* = 6) / Control (*n* = 5) ratio (Ratio variability [%])	IgAVN-II (*n* = 6) / Control (*n* = 5) ratio (Ratio variability [%])
4235275		Talin 1	9	18	100 **		100 **	100 **
4758822		Nephrin	4	5	1.01 (95.98)	0.87 (59.76)	0.82 (49.93)	0.90 (20.54)
7657615		Podocin	6	2	2.64 (78.71)	1.83 (87.04)	3.10 (83.40)	3.80 (70.67)
1034591669		Zo-1	5	8	0.89 (81.11)	0.70 (76.53)	1.38 (86.88)	1.03 (99.74)
5453599		CapZA2	9	2	0.51 (19.82)	0.63 (15.37)	0.68 (5.62)	0.81 (20.25)
62414289		Vimentin	79	56	0.98 (89.42)	0.83 (102.95)	1.15 (100.59)	1.24 (95.28)
578810794		Synaptopodin	13	10	1.72 (61.40)	1.04 (63.53)	1.33 (59.27)	1.55 (63.72)
12025678		α-Actinin-4	50	38	1.42 (81.14)	0.83 (92.02)	1.26 (79.98)	1.24 (87.68)
12667788		Myosin-9	16	26	0.99 (79.27)	0.82 (83.75)	0.89 (86.32)	0.90 (84.35)
219520307		Podocalyxin	9	5	2.25 (81.92)	1.21 (81.52)	2.40 (81.50)	1.81 (81.90)
119615053		Integrin α3	4	4	1.26 (69.82)	0.80 (83.25)	1.17 (70.16)	1.15 (70.64)

CapZA2: F-actin capping protein α2; DB: database; IgAN: IgA nephropathy; IgAVN: IgA vasculitis with nephritis; NCBInr: National Center for Biotechnology information non-redundant; Zo-1: zonula occludens-1

**P < 0.01

**Table 6:** List of glomerular basement membrane proteins

Accession ID NCBInr DB	Accession ID GenBank DB	Protein name	Sequence coverage (%)	Peptide match (*n*)	IgAN-I (*n* = 6) / Control (*n* = 5) ratio (Ratio variability [%])	IgAN-II (*n* = 6) / Control (*n* = 5) ratio (Ratio variability [%])	IgAVN-I (*n* = 6) / Control (*n* = 5) ratio (Ratio variability [%])	IgAVN-II (*n* = 6) / Control (*n* = 5) ratio (Ratio variability [%])
125987809		COL4 α1	6	5	0.27 (87.87)	0.28 (109.92)	0.12 ** (53.43)	0.60 (129.11)
116256354		COL4 α2	7	9	2.14 (55.82)	2.13 (77.60)	1.40 (71.10)	1.55 (49.45)
119591260		COL4 α3	7	6	2.20 (108.21)	0.82 (112.25)	1.57 (113.08)	1.71 (114.47)
116256356		COL4 α4	4	4	1.50 (57.26)	1.41 (76.00)	1.14 (77.27)	1.62 (58.07)
1034673478		COL4 α5	4	4	2.02 (76.80)	1.35 (72.18)	1.48 (92.65)	2.16 (80.63)
20147503		Laminin α5	19	58	1.72 (111.36)	2.25 (100.34)	1.40 (108.82)	1.85 (93.85)
1103585		Laminin β2	28	44	1.27 (84.02)	0.91 (83.36)	1.06 (85.04)	1.09 (85.79)
145309326		Laminin γ1	24	32	1.32 (72.95)	1.02 (78.48)	1.07 (86.85)	1.11 (81.63)
115298674		Nidogen-1	19	24	2.10 (45.61)	1.57 (81.36)	1.87 (60.30)	2.15 (46.04)
530360311		Agrin	27	40	0.99 (78.54)	0.64 (82.89)	0.60 (93.13)	0.72 (82.41)
11602963		HSPG perlecan	17	54	1.31 (76.31)	1.19 (81.45)	1.08 (84.44)	1.15 (73.35)
34364820		Fibronectin	22	38	2.42 (94.55)	2.95 (100.41)	2.59 (94.34)	2.47 (97.14)

COL4: type IV collagen; DB: database; HSPG: heparan sulfate proteoglycan; IgAN: IgA nephropathy; IgAVN: IgA vasculitis with nephritis; NCBInr: National Center for Biotechnology information non-redundant

**P < 0.01

**Table 7:** List of extracellular matrix-associated proteins

Accession ID NCBInr DB	Accession ID GenBank DB	Protein name	Sequence coverage (%)	Peptide match (*n*)	IgAN-I (*n* = 6) / Control (*n* = 5) ratio (Ratio variability [%])	IgAN-II (*n* = 6) / Control (*n* = 5) ratio (Ratio variability [%])	IgAVN-I (*n* = 6) / Control (*n* = 5) ratio (Ratio variability [%])	IgAVN-II (*n* = 6) / Control (*n* = 5) ratio (Ratio variability [%])
4503689		Fibrinogen α	14	11	3.42 (88.22)	9.17 ** (92.08)	6.42 (91.47)	6.11 (82.12)
70906435		Fibrinogen β	10	4	41.83 ** (37.42)	44.84 ** (56.21)	54.46 ** (42.26)	43.68 ** (52.45)
182440		Fibrinogen γ	10	3	100 **	100 **	100 **	100 **
193787687	AK094581.1	βIG-H3	5	3	5.50 (85.79)	9.24 ** (56.45)	8.15 * (43.46)	5.09 (44.75)
57546919		Hornerin	24	23	0.13 * (111.3)	0.20 ** (134.16)	0.23 (105.85)	0.11 ** (113.00)

βIG-H3: transforming growth factor-β inducible gene-h3; DB: database; IgAN: IgA nephropathy; IgAVN: IgA vasculitis with nephritis; NCBInr: National Center for Biotechnology information non-redundant

*P < 0.05, **P < 0.01

Igs are summarized in Table 3. The Ig α1, α2, γ1, γ2, γ3, γ4, and µ heavy chains, Ig κ and λ light chains, and Ig joining chain (J chain) [18] were detected, while the polymeric Ig receptor/secretory component (pIgR/SC) [18] was not. The different accession numbers for the Ig α1, γ1, and µ heavy chains are listed in Supplementary Tables S3 and S4 (Additional files 3 and 4). In these chains, the peptides detected and used to compare protein abundance are summarized in Supplementary Tables S5, S6, and S7 (Additional files 5, 6, and 7). Among Igs, significantly higher levels of the Ig α1, α2, γ1, γ2, γ4, and µ heavy chains and Ig κ and λ light chains were noted in the IgAN and IgAVN subgroups than in the control group. The level of the Ig J chain was also higher (> 2.3 fold) in the IgAN and IgAVN subgroups than in the control group.

Complements and complement-regulating proteins are summarized in Table 4. Complements C3, C4A, C5, C8γ, and C9 were detected. Among them, the levels of C3, C4A, C5, and C9 were higher (> 2.6 fold) in the IgAN and IgAVN subgroups than in the control group. Complement factor H-related proteins (CFHR) 1 and 5, vitronectin, clusterin, the C4b-binding protein α chain, and complement receptor 1 (CR1) were also detected. Among them, CFHR1, CFHR5, and vitronectin levels were higher (> 2.8 fold) in the IgAN and IgAVN subgroups than in the control group, whereas CR1 levels were lower (< 0.6 fold) in the IgAN and IgAVN subgroups than in the control group. The complement C3 peptides detected were further analyzed. Peptides with significantly higher levels in the IgAN and IgAVN subgroups than in the control group were mainly derived from the MG2, MG3, MG6β, TED, and CTC (C345c) domains, but not from the ANA domain containing C3a anaphylatoxin (Additional file 8: Table S8) [19, 20].

The podocyte-associated proteins detected [21, 22], including talin 1 [23], nephrin, podocin, zonula occludens-1 (Zo-1), F-actin capping protein α2 (CapZA2) [24], vimentin [25], synaptopodin, α-actinin-4, myosin-9, podocalyxin, and integrin α3, are summarized in Table 5. Among them, talin 1 levels were significantly higher in the IgAN-I subgroup and IgAVN group than in the control group, but were not detected in the IgAN-II subgroup. Podocin levels were higher (> 2.6 fold) in the IgAN-I subgroup and IgAVN group than in the control group.

The GBM proteins detected [12, 26], including type IV collagen (COL4) α chains, laminin α, β, and γ chains, nidogen-1, agrin, heparan sulfate proteoglycan perlecan, and fibronectin, are summarized in Table 6. Among them, fibronectin levels were higher (> 2.4 fold) in the IgAN and IgAVN subgroups than in the control group, whereas COL4 α1 chain levels were lower (≤ 0.6 fold) in the IgAN and IgAVN subgroups than in the control group.

The ECM-associated proteins detected [12], including fibrinogen, transforming growth factor-β inducible gene-h3 (βIG-H3), and hornerin, are summarized in Table 7. Among them, the levels of the fibrinogen α, β, and γ chains and βIG-H3 were higher (> 3.4 fold) in the IgAN and IgAVN subgroups than in the control group, whereas those of hornerin were lower (< 0.3).

Table 8 summarizes proteins with abundance that significantly differed between the IgAN and IgAVN groups, the IgAN-I and -II subgroups, the IgAVN-I and -II subgroups, and the IgAVN-III and -IV subgroups. Significantly different levels of C9, CFHR1, podocin, Zo-1, CapZA2, and vimentin were observed between the IgAN and IgAVN groups. Furthermore, CR1, podocin, synaptopodin, α-actinin-4, podocalyxin, COL4 α5 chain, and laminin β2 chain levels significantly differed between the IgAN-I and -II subgroups. Podocin, synaptopodin, and laminin β2 chain levels also significantly differed between the IgAVN-III and -IV subgroups.

**Table 8:** List of proteins with abundance that significantly differed between groups

Accession ID NCBInr DB	Protein name	IgAN total vs. IgAVN total		IgAN-I vs. IgAN-II		IgAVN-I vs. IgAVN-II		IgAVN-III vs. IgAVN-IV	
		Fold change	*P* value	Fold change	*P* value	Fold change	*P* value	Fold change	*P* value
119576392	C9	2.26	0.043 *	1.82	0.228	1.75	0.203	1.14	0.811
183763	CFHR1	3.48	0.028 *	0.79	0.689	1.07	0.898	0.65	0.427
809019	CR1	0.68	0.273	7.92	0.002 **	0.91	0.838	3.77	0.058
7657615	Podocin	0.63	0.007 **	2.04	0.017 *	1.08	0.618	1.55	0.017 *
1034591669	Zo-1	0.43	0.001 **	1.91	0.064	0.99	0.984	1.63	0.163
5453599	CapZA2	0.64	0.003 **	1.26	0.464	0.85	0.130	0.77	0.008 **
62414289	Vimentin	0.74	0.003 **	1.19	0.367	0.98	0.815	1.03	0.738
578810794	Synaptopodin	0.82	0.278	2.42	0.009 **	0.97	0.833	1.42	0.025 *
12025678	α-Actinin-4	0.86	0.264	1.59	0.027 *	0.96	0.775	1.26	0.202
219520307	Podocalyxin	0.82	0.300	2.36	0.001 **	1.22	0.432	1.51	0.184
1034673478	COL4 α5	0.88	0.490	1.87	0.034 *	0.86	0.483	1.62	0.072
1103585	Laminin β2	0.93	0.569	1.54	0.032 *	0.98	0.911	1.54	0.035 *

CapZA2: F-actin capping protein α2; CFHR: complement factor H-related protein; COL4: type IV collagen; CR1: complement receptor 1; DB: database; IgAN: IgA nephropathy; IgAVN: IgA vasculitis with nephritis; NCBInr: National Center for Biotechnology information non-redundant; Zo-1: zonula occludens-1

*P < 0.05, **P < 0.01

### Immunohistochemistry

An immunohistochemical study was performed on biopsy specimens from 3 IgAN patients without NS and 3 IgAN patients with NS. Images of talin 1 staining on 3 glomeruli and the surrounding tubules in each patient in the non-NS and NS groups are shown in Supplementary Figures S1 and S2 (Additional files 9 and 10), respectively.

**Fig. 2:**
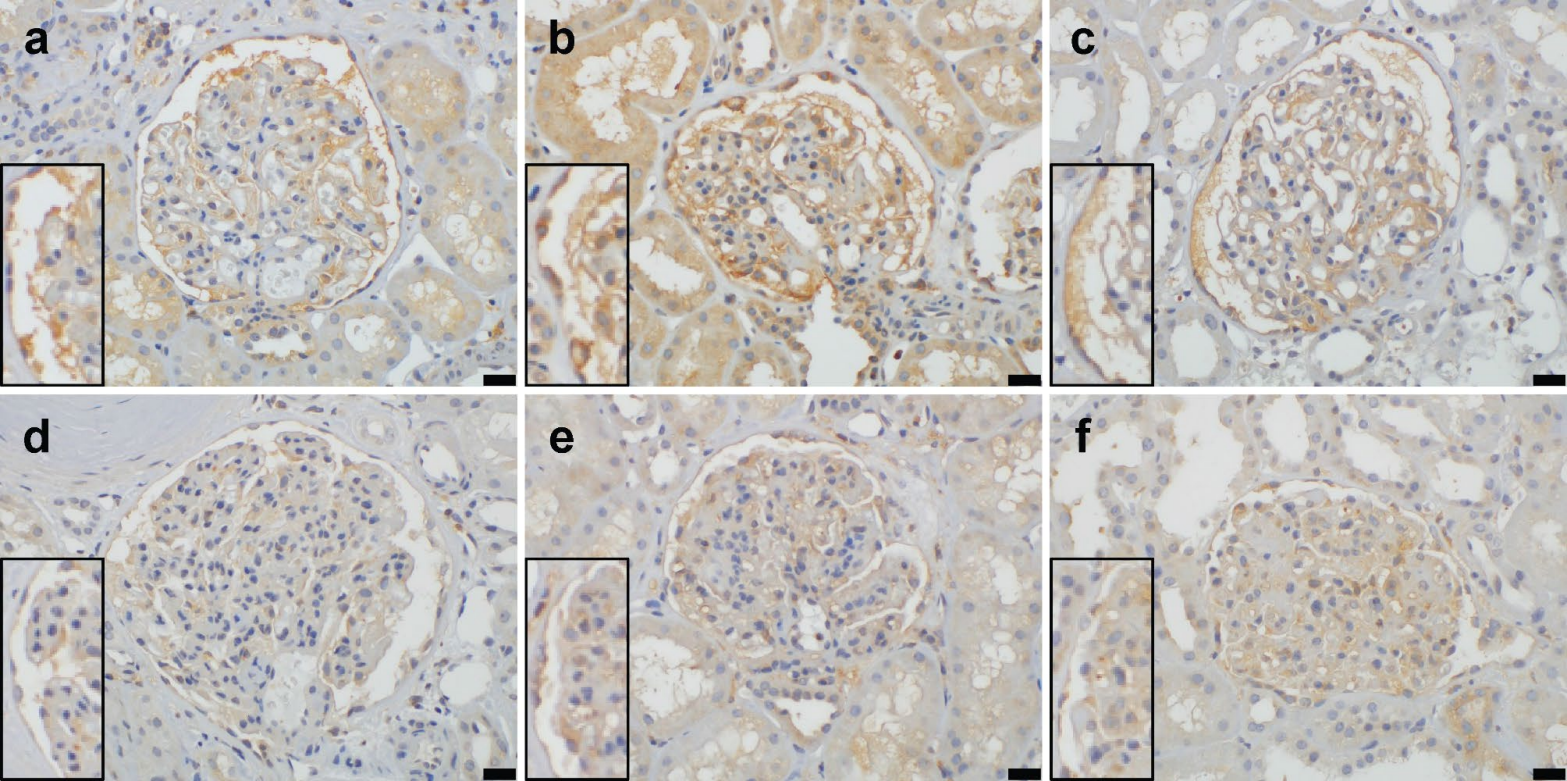
Immunohistochemical staining for talin 1. **a-c** Three IgAN patients without NS. Glomerular talin 1 staining is observed in parietal epithelial cells and podocytes, along capillary walls, and in the urinary space (probable cell debris of detached parietal epithelial cells and podocytes). Staining for talin 1 is also detected in proximal tubular epithelial cells. **d-f** Three IgAN patients with NS. Severe mesangial expansion is observed. Staining for talin 1 in parietal epithelial cells and podocytes, along capillary walls, and in the urinary space is less intense than that in panels **a-c**. Enlarged images are inserted at the lower left. Bars represent 20 μm. Original magnification of each image: ×400

In IgAN patients without NS (Figs. 2a, 2b, and 2c), glomerular talin 1 staining was observed in parietal epithelial cells and podocytes, along capillary walls, and in the urinary space (probable cell debris of detached parietal epithelial cells and podocytes). Talin 1 staining was also detected in proximal tubular epithelial cells, suggesting the reabsorption of talin 1 derived from detached parietal epithelial cells and podocytes.

Severe mesangial expansion was observed in IgAN patients with NS (Figs. 2d, 2e, and 2f). Staining for talin 1 in parietal epithelial cells and podocytes, along capillary walls, and in the urinary space was less intense than in IgAN patients without NS.

Glomerular T1S-PARs were slightly higher in patients without NS (14.50 ± 12.11%) than in patients with NS (4.93 ± 2.60%) (*P* = 0.151).

## Discussion

In 1978, Nakamoto et al. [27] reported indistinguishable immunohistological features between IgAN and IgAVN. These 2 diseases have since been considered to be closely related [1, 3–5]. A recent clinicopathological study by Sugiyama et al. [28] suggested that adult-onset IgAVN presents as acute glomerular inflammatory lesions with endothelial injury, while IgAN develops as chronic progressive mesangial lesions. Fang et al. [14] recently compared urinary proteomic profiles between pediatric patients with IgAN and IgAVN and healthy controls. They found that the proteins expressed in the IgAN and IgAVN groups were mainly involved in the immune system, cell proliferation, and signaling. These findings indicated that many common pathways play important roles in the progression of IgAN and IgAVN in children.

In the present study, we performed a comparative proteomic analysis of glomerular proteins extracted from the laser microdissected glomeruli of patients with IgAN and adult-onset IgAVN and control cases (time 0 transplant biopsies). A principal component analysis demonstrated a clear separation between the IgAN and IgAVN groups and the control group as well as an overlapping distribution between the IgAN and IgAVN groups (Fig. 1). More than 850 proteins with high confidence were identified, and 546 proteins that were matched with ≥ 2 peptides were selected for a more precise analysis. We then listed proteins, such as Igs, complement proteins, complement-regulating proteins, podocyte-associated proteins, GBM proteins, and ECM-associated proteins. After analyzing the abundance of these proteins, proteins in each IgAN and IgAVN subgroup were compared with those in the control group. We also compared the abundance of these proteins between the IgAN and IgAVN groups, the IgAN-I and -II subgroups, the IgAVN-I and -II subgroups, and the IgAVN-III and -IV subgroups.

Regarding Igs (Table 3), the Ig α1, α2, γ1, γ2, γ3, γ4, and µ heavy chains, Ig κ and λ light chains, and Ig J chain were detected. The levels of these Ig heavy and light chains were significantly higher in the IgAN and IgAVN groups than in the control group, except for the Ig γ3 heavy chain. In a proteomic study on glomerular proteins in IgAN by Kawata et al. [13], increased levels of the Ig α1, γ1, γ2, and µ heavy chains were also observed in their patients. The mesangial IgA of IgAN is exclusively of the IgA1 subclass Gd-IgA1 [1], and Gd-IgA1-specific IgG is the most frequent of the IgG2 subclass [29]. Our immunofluorescence study showed positive staining for IgA1 and negative staining for IgA2 in most patients with IgAN and IgAVN. This result is consistent with the findings by Conley et al. [30]. In their study, the staining intensity of the Ig J chain, a component of polymeric IgM and IgA [18], correlated with that of IgM, but not IgA. Based on these findings, most of the Ig J chain detected in the present study appeared to originate from polymeric IgM. In addition, pIgR/SC, which is necessary for secretory IgA formation [18], was not detected in glomerular extracts from patients with IgAN and IgAVN. The present results indicate that the co-deposits of all IgA and IgG subclasses and IgM were detectable by a highly sensitive nLC-MS/MS analysis of the IgAN and IgAVN subgroups, and suggest shared mechanisms for glomerular Ig deposition in the 2 diseases.

Regarding complement proteins and complement-regulating proteins (Table 4), higher levels (> 2.6 fold) of C3, C4A, C5, C9, CFHR1, CFHR5, and vitronectin and lower levels (< 0.6 fold) of CR1 were observed in the IgAN and IgAVN subgroups than in the control group. These results are consistent with the findings of a proteomic study on glomerular proteins in IgAN by Paunas et al. [11], except that C1 subcomponents were not identified in the present study. In an immunohistochemical study by Moll et al. [31], decreased glomerular staining for CR1 was also observed in patients with IgAN and IgAVN. Since CR1 is an inhibitor of the alternative pathway [11], a decrease in CR1 may enhance the detrimental effects of complement activation in IgAN and IgAVN. The present results and previous findings by Paunas et al. [11] and Moll et al. [31] suggest common activation mechanisms in the complement pathways in IgAN and IgAVN. We also found that glomerular C9 and CFHR1 were significantly more abundant in the IgAN group than in the IgAVN group, and that CR1 levels were significantly lower in the IgAN-II subgroup than in the IgAN-I subgroup (Table 8). As suggested by a growing body of evidence, glomerular complement activation is strongly associated with IgAN and may be the dominant driver of glomerular injury leading to proteinuria [32]. Since the mesangial co-deposition of IgA and complement C3 is characteristic of IgAN, we further analyzed the C3 peptides detected at significantly higher levels in the IgAN and IgAVN subgroups than in the control group (Additional file 8: Table S8). The results obtained suggested the presence of C3 breakdown products [19, 20] in glomeruli in IgAN and IgAVN; however, it was not possible to identify the types of fragments deposited.

Limited information is currently available on changes to the expression of podocyte-associated molecules in acquired human kidney diseases [33]. The main podocyte-associated proteins were detected in our proteomic analysis (Table 5). Among them, significantly higher levels of talin 1, a critical protein for podocyte cytoskeletal stability [23], were observed in the IgAN-I subgroup and IgAVN group than in the control group. Manso et al. [34] found that mechanical stress causing cardiac hypertrophy induced the expression of talin 1 in cardiac myocytes, and suggested its unique role in the molecular responses of the myocardium to stress. Based on these findings, increased talin 1 levels in the IgAN-I subgroup and IgAVN group may result from stress responses in injured podocytes. In contrast, talin 1 was not detected in the IgA-II subgroup with NS. This was supported by immunohistochemical findings (Fig. 2). These results may be related to advanced podocyte injury and massive proteinuria in IgAN with NS. Tian et al. [23] showed that mice lacking talin 1 specifically in podocytes exhibited severe proteinuria. In addition, our immunohistochemical findings indicated the potential of urinary talin 1 levels as a useful biomarker for the early detection of podocytopathy [2] (Fig. 2). We also found that podocin levels were higher (> 2.6 fold) in the IgAN-I subgroup and IgAVN group than in the control group (Table 5). Podocin is known to be involved in the response of podocytes to mechanical forces [35]. Therefore, up-regulated podocin may play a role in the structural maintenance of stressed podocytes in IgAN without NS and IgAVN. Moreover, the abundance of podocin was significantly less in the IgAN-II subgroup than in the IgAN-I subgroup as well as in the IgAVN-IV subgroup than in the IgAVN-III subgroup (Table 8). Podocin is a crucial protein in the slit diaphragm, a size-selective barrier between 2 podocyte foot processes [22]. Decreased podocin synthesis may impair glomerular filtration and induce massive proteinuria in IgAN and IgAVN patients with NS.

We also found significant changes in other podocyte-associated proteins that are important for the maintenance of the glomerular filtration barrier in IgAN and IgAVN. In comparisons with the IgAVN group, the levels of Zo-1, CapZA2, and vimentin, which play crucial roles in the formation and maintenance of the slit diaphragm and podocyte foot processes [22, 24, 25], significantly decreased in the IgAN group (Table 8). During the chronic phase of IgAN, the abundance of these proteins may have been reduced due to podocyte injury [2, 21]. In comparisons with the IgAN-I subgroup, the abundance of synaptopodin, α-actinin-4, and podocalyxin, which are actin cytoskeleton-associated proteins involved in maintaining podocyte foot processes [22], was significantly less in the IgAN-II subgroup (Table 8). The abundance of synaptopodin was also significantly less in the IgAVN-IV subgroup than in the IgAVN-III subgroup (Table 8). Since mutations affecting the genes encoding synaptopodin and α-actinin-4 cause proteinuric glomerular diseases [22], the present results suggest changes in the expression of these proteins in injured podocytes as the underlying cause of massive proteinuria in IgAN and IgAVN patients with NS. As a related observation, an electron microscopic study on IgAN patients by Lee et al. [36] showed that proteinuria positively correlated with the severity of foot process effacement, one of the general changes in shape within injured podocytes [37].

Main GBM proteins were also detected in our proteomic analysis (Table 6). Among them, fibronectin levels were higher (> 2.4 fold) in the IgAN and IgAVN subgroups than in the control group. This result is consistent with the findings of a proteomic analysis of glomerular proteins in IgAN by Paunas et al. [12]. On the other hand, COL4 α1 chain levels were lower (≤ 0.6 fold) in the IgAN and IgAVN subgroups than in the control group. This result is inconsistent with the findings reported by Paunas et al. [12]. The reason for this discrepancy currently remains unclear. Since the mature GBM is composed of the COL4 α3, α4, and α5 chains [26], decreased COL4 α1 chain levels may play a minor role in GBM assembly in IgAN and IgAVN, which is consistent with our previous findings on MN [8]. We also noted that the abundance of the COL4 α5 chain and laminin β2 chain was significantly less in the IgAN-II subgroup than in the IgAN-I subgroup (Table 8). Furthermore, the abundance of the laminin β2 chain was significantly less in the IgAVN-IV subgroup than in the IgAVN-III subgroup (Table 8). These results indicate that impaired GBM contributes to the development of massive proteinuria in IgAN and IgAVN patients with NS. Mutations in the genes encoding the COL4 α5 chain and laminin β2 chain have been implicated in the pathogenesis of proteinuric glomerular diseases [38, 39].

Various ECM-associated proteins were detected in our proteomic analysis (Table 7). Consistent with the findings reported by Paunas et al. [12], the levels of fibrinogen chains and βIG-H3 were higher (> 3.4 fold) in the IgAN and IgAVN subgroups than in the control group, whereas those of hornerin were lower (< 0.3 fold). Katafuchi et al. [40] and Wang et al. [41] evaluated the significance of glomerular fibrinogen deposition in IgAN or IgAVN. Katafuchi et al. [40] suggested that the deposition of IgA together with IgG, C3, and fibrinogen induced acute inflammatory injury in IgAN. Wang et al. [41] indicated that the deposition of fibrinogen plays an important role in that of IgA and IgG as well as tissue injury in IgAVN. These findings and the present results collectively suggest similar mechanisms for the glomerular deposition of Ig together with fibrinogen in IgAN and IgAVN. Limited information is currently available on the roles of βIG-H3 and hornerin in IgAN and/or IgAVN. Zhou et al. [42] recently identified the gene encoding βIG-H3 (*TGFBI* [12]) as a susceptibility gene involved in IgAN among Han Chinese. Hornerin is a component of the cutaneous innate antimicrobial defense system [43]; however, its role in glomeruli remains unknown.

As described above, the abundance of several proteins involved in the complement system and glomerular filtration barrier significantly differed between the IgAN-I and -II subgroups. The abundance of some proteins involved in the glomerular filtration barrier also significantly differed between the IgAVN-III and -IV subgroups. These results suggest shared pathogenetic mechanisms for the development of massive proteinuria in the 2 diseases. On the other hand, these differences were not observed between the IgAVN-I and -II subgroups. Crescent formation requires glomerular capillary injury causing GBM rupture, which triggers plasmatic coagulation within the urinary space. Coagulation factors and other mitogenic signals then induce parietal epithelial cell hyperplasia followed by cellular crescent formation [44]. In the present study, glomerular tufts excluding crescentic lesions were microdissected, as described by Paunas et al. [12], and most IgAVN patients had ISKDC grade III glomerular lesions. Therefore, difficulties are associated with detecting significant differences in the abundance of proteins within glomerular tufts between the IgAVN subgroups. Further studies are needed to elucidate the complex molecular mechanisms underlying vascular endothelial injury in IgAV [45].

In the present study, it was difficult to enroll a larger number of IgAN patients with NS and IgAVN patients because of the small number who underwent biopsy. In IgAN patients with NS, it was also challenging to prepare adequate biopsy specimens for the laser microdissection of glomeruli and immunohistochemical studies because of advanced glomerulosclerosis. Despite the power of proteomic profiling, there are several limitations that need to be addressed. We performed nLC-MS/MS on a small number of glomerulus samples. Pediatric patients with IgAVN were not included. Therefore, further analyses of a larger sample size are required to increase the accuracy of our study. Furthermore, the present results on Japanese patients with IgAN and IgAVN need to be assessed in other ethnic cohorts because a role for genetic factors in the pathogenesis of these 2 diseases has been suggested [1, 3]. Moreover, significant differences were observed in clinical features between the IgAN and IgAVN subgroups. The time from clinical presentation (chance proteinuria and/or hematuria) to biopsy was delayed in the IgAN-I subgroup. In addition, there was a significant age difference between IgAN patients with and without NS in immunohistochemical validation studies. The possibility that nephrosis is caused by age rather than by the IgAN pathology cannot be ruled out. Another limitation is that controls of glomerular diseases, such as minimal change nephrotic syndrome (MCNS), were not included. A comparison of data on MCNS patients with those on nephrotic IgAN and IgAVN patients may provide important insights into differences in the onset mechanisms between IgAN- and IgAVN-induced nephrosis and MCNS.

## Conclusions

This is the first study to perform a comparative proteomic analysis of glomerular proteins extracted from laser microdissected glomeruli in IgAN and IgAVN. The results obtained suggest shared molecular mechanisms for glomerular injury in these 2 diseases, except for enhanced glomerular complement activation in IgAN. Differences in the protein abundance of some podocyte-associated proteins and GBM proteins between patients with and without NS in IgAN and IgAVN may be associated with the severity of proteinuria. The present results provide a more detailed understanding of the pathophysiology of IgAN and IgAVN.

## Electronic supplementary material

Below is the link to the electronic supplementary material.

**Additional file 1: table S1** Clinical characteristics of IgAN patients included in the immunohistochemical study.

**Additional file 2: table S2** Pathological characteristics of IgAN patients included in the immunohistochemical study.

**Additional file 3: table S3** Summary of proteomics data (Proteins).

**Additional file 4: table S4** Summary of proteomics data (Peptides).

**Additional file 5: table S5** List of IgA1 peptides.

**Additional file 6: table S6** List of IgG1 peptides.

**Additional file 7: table S7** List of IgM peptides.

**Additional file 8: table S8** List of complement C3 peptides.

**Additional file 9: figure S1** Immunohistochemical staining for talin 1 in IgAN patients without NS. **a-c**: A 35-year-old male; **d-f**: a 19-year-old female; **g-i**: a 39-year-old female. Bars represent 20 μm. Original magnification of each image: ×400.

**Additional file 10: figure S2** Immunohistochemical staining for talin 1 in IgAN patients with NS. **a-c**: An 80-year-old female; **d-f**: a 62-year-old male; **g-i**: a 82-year-old female. Bars represent 20 μm. Original magnification of each image: ×400.

## Data Availability

Please contact the corresponding author for data requests.

## Abbreviations

βIG-H3: Transforming growth factor-β inducible gene-h3
CapZA2: F-actin capping protein α2
CFHR: Complement factor H-related protein
COL4: Type IV collagen
Cr: Creatinine
CR1: Complement receptor 1
ECM: Extracellular matrix
GBM: Glomerular basement membrane
Gd-IgA1: Galactose-deficient IgA1
Ig: Immunoglobulin
IgAN: IgA nephropathy
IgAV: IgA vasculitis
IgAVN: IgA vasculitis with nephritis
ISKDC: International Study of Kidney Disease in Children
J chain: Joining chain
MN: Membranous nephropathy
NCBInr: National Center for Biotechnology Information non-redundant database
nLC-MS/MS: Nano-liquid chromatography-tandem mass spectrometry
NS: Nephrotic syndrome
pIgR/SC: Polymeric immunoglobulin receptor/secretory component
Zo-1: Zona occludens-1

## References

[1] Rajasekaran A, Julian BA, Rizk DV (2021). IgA nephropathy: an interesting autoimmune kidney disease. Am J Med Sci.

[2] Trimarchi H, Coppo R (2019). Podocytopathy in the mesangial proliferative immunoglobulin a nephropathy: new insights into the mechanisms of damage and progression. Nephrol Dial Transplant.

[3] Hastings MC, Rizk DV, Kiryluk K, Nelson R, Zahr RS, Novak J (2022). IgA vasculitis with nephritis: update of pathogenesis with clinical implications. Pediatr Nephrol.

[4] Suzuki H, Yasutake J, Makita Y, Tanbo Y, Yamasaki K, Sofue T (2018). IgA nephropathy and IgA vasculitis with nephritis have a shared feature involving galactose-deficient IgA1-oriented pathogenesis. Kidney Int.

[5] Pillebout E (2021). IgA vasculitis and IgA nephropathy: same disease?. J Clin Med.

[6] Saito A, Komatsuda A, Kaga H, Sato R, Togashi M, Okuyama S (2016). Different expression patterns of toll-like receptor mRNAs in blood mononuclear cells of IgA nephropathy and IgA vasculitis with nephritis. Tohoku J Exp Med.

[7] Miyakawa R, Sato A, Matsuda Y, Saito A, Abe F, Matsumura H (2020). Comparative proteomic analysis of renal proteins from IgA nephropathy model mice and control mice. Clin Exp Nephrol.

[8] Kaga H, Matsumura H, Suzuki T, Dohmae N, Odaka M, Komatsuda A (2022). Comparative proteomic analysis of glomerular proteins in primary and bucillamine-induced membranous nephropathy. Clin Proteomics.

[9] Taherkhani A, Farrokhi Yekta R, Mohseni M, Saidijam M, Arefi Oskouie A (2019). Chronic kidney disease: a review of proteomic and metabolomic approaches to membranous glomerulonephritis, focal segmental glomerulosclerosis, and IgA nephropathy biomarkers. Proteome Sci.

[10] Fang X, Wu H, Lu M, Cao Y, Wang R, Wang M (2020). Urinary proteomics of Henoch-Schönlein purpura nephritis in children using liquid chromatography-tandem mass spectrometry. Clin Proteomics.

[11] Paunas TIF, Finne K, Leh S, Marti HP, Mollnes TE, Berven F (2017). Glomerular abundance of complement proteins characterized by proteomic analysis of laser-captured microdissected glomeruli associates with progressive disease in IgA nephropathy. Clin Proteomics.

[12] Paunas FTI, Finne K, Leh S, Osman TA, Marti HP, Berven F (2019). Characterization of glomerular extracellular matrix in IgA nephropathy by proteomic analysis of laser-captured microdissected glomeruli. BMC Nephrol.

[13] Kawata N, Kang D, Aiuchi T, Obama T, Yoshitake O, Shibata T (2020). Proteomics of human glomerulonephritis by laser microdissection and liquid chromatography-tandem mass spectrometry. Nephrology (Carlton).

[14] Fang X, Lu M, Xia Z, Gao C, Cao Y, Wang R (2021). Use of liquid chromatography-tandem mass spectrometry to perform urinary proteomic analysis of children with IgA nephropathy and Henoch-Schönlein purpura nephritis. J Proteomics.

[15] Ozen S, Pistorio A, Iusan SM, Bakkaloglu A, Herlin T, Brik R (2010). EULAR/PRINTO/PRES criteria for Henoch-Schönlein purpura, childhood polyarteritis nodosa, childhood Wegener granulomatosis and childhood Takayasu arteritis: Ankara 2008. Part II: final classification criteria. Ann Rheum Dis.

[16] Schindelin J, Arganda-Carreras I, Frise E, Kaynig V, Longair M, Pietzsch T (2012). Fiji: an open-source platform for biological-image analysis. Nat Methods.

[17] Zhao L, Cong X, Zhai L, Hu H, Xu JY, Zhao W (2020). Comparative evaluation of label-free quantification strategies. J Proteomics.

[18] Wines BD, Hogarth PM (2006). IgA receptors in health and disease. Tissue Antigens.

[19] Papanastasiou M, Koutsogiannaki S, Sarigiannis Y, Geisbrecht BV, Ricklin D, Lambris JD (2017). Structural implications for the formation and function of the complement effector protein iC3b. J Immunol.

[20] Geisbrecht BV, Lambris JD, Gros P (2022). Complement component C3: a structural perspective and potential therapeutic implications. Semin Immunol.

[21] Kopp JB, Anders HJ, Susztak K, Podestà MA, Remuzzi G, Hildebrandt F (2020). Podocytopathies. Nat Rev Dis Primers.

[22] Blaine J, Dylewski J (2020). Regulation of the actin cytoskeleton in podocytes. Cells.

[23] Tian X, Kim JJ, Monkley SM, Gotoh N, Nandez R, Soda K (2014). Podocyte-associated talin1 is critical for glomerular filtration barrier maintenance. J Clin Invest.

[24] van Duijn TJ, Anthony EC, Hensbergen PJ, Deelder AM, Hordijk PL (2010). Rac1 recruits the adapter protein CMS/CD2AP to cell-cell contacts. J Biol Chem.

[25] Ge X, Zhang T, Yu X, Muwonge AN, Anandakrishnan N, Wong NJ (2020). LIM-nebulette reinforces podocyte structural integrity by linking actin and vimentin filaments. J Am Soc Nephrol.

[26] Naylor RW, Morais MRPT, Lennon R (2021). Complexities of the glomerular basement membrane. Nat Rev Nephrol.

[27] Nakamoto Y, Asano Y, Dohi K, Fujioka M, Iida H, Kida H (1978). Primary IgA glomerulonephritis and Schönlein-Henoch purpura nephritis: clinicopathological and immunohistological characteristics. Q J Med.

[28] Sugiyama M, Wada Y, Kanazawa N, Tachibana S, Suzuki T, Matsumoto K (2020). A cross-sectional analysis of clinicopathologic similarities and differences between Henoch-Schönlein purpura nephritis and IgA nephropathy. PLoS One.

[29] Suzuki H, Moldoveanu Z, Hall S, Brown R, Julian BA, Wyatt RJ (2007). IgA nephropathy: characterization of IgG antibodies specific for galactose-deficient IgA1. Contrib Nephrol.

[30] Conley ME, Cooper MD, Michael AF (1980). Selective deposition of immunoglobulin A_1_ in immunoglobulin A nephropathy, anaphylactoid purpura nephritis, and systemic lupus erythematosus. J Clin Invest.

[31] Moll S, Miot S, Sadallah S, Gudat F, Mihatsch MJ, Schifferli JA (2001). No complement receptor 1 stumps on podocytes in human glomerulopathies. Kidney Int.

[32] Tortajada A, Gutierrez E, Pickering MC, Praga Terente M, Medjeral-Thomas N (2019). The role of complement in IgA nephropathy. Mol Immunol.

[33] Koop K, Eikmans M, Baelde HJ, Kawachi H, de Heer E, Paul LC (2003). Expression of podocyte-associated molecules in acquired human kidney diseases. J Am Soc Nephrol.

[34] Manso AM, Li R, Monkley SJ, Cruz NM, Ong S, Lao DH (2013). Talin1 has unique expression *versus* talin 2 in the heart and modifies the hypertrophic response to pressure overload. J Biol Chem.

[35] Endlich K, Kliewe F, Endlich N (2017). Stressed podocytes––mechanical forces, sensors, signaling and response. Pflugers Arch.

[36] Lee JH, Jang SH, Cho NJ, Heo NH, Gil HW, Lee EY (2020). Severity of foot process effacement is associated with proteinuria in patients with IgA nephropathy. Kidney Res Clin Pract.

[37] Kriz W, Shirato I, Nagata M, LeHir M, Lemley KV (2013). The podocyte’s response to stress: the enigma of foot process effacement. Am J Physiol Renal Physiol.

[38] Gast C, Pengelly RJ, Lyon M, Bunyan DJ, Seaby EG, Graham N (2016). Collagen (*COL4A*) mutations are the most frequent mutations underlying adult focal segmental glomerulosclerosis. Nephrol Dial Transplant.

[39] Chen YM, Kikkawa Y, Miner JH (2011). A missense *LAMB2* mutation causes congenital nephrotic syndrome by impairing laminin secretion. J Am Soc Nephrol.

[40] Katafuchi R, Nagae H, Masutani K, Tsuruya K, Mitsuiki K (2019). Comprehensive evaluation of the significance of immunofluorescent findings on clinicopathological features in IgA nephropathy. Clin Exp Nephrol.

[41] Wang F, Huang L, Tang H, Li X, Zhu X, Wang X (2018). Significance of glomerular fibrinogen deposition in children with Henoch-Schönlein purpura nephritis. Ital J Pediatr.

[42] Zhou XJ, Tsoi LC, Hu Y, Patrick MT, He K, Berthier CC (2021). Exome chip analyses and genetic risk for IgA nephropathy among Han Chinese. Clin J Am Soc Nephrol.

[43] Christophers E, Schröder JM (2022). Evolution of innate defense in human skin. Exp Dermatol.

[44] Anguiano L, Kain R, Anders HJ (2020). The glomerular crescent: triggers, evolution, resolution, and implications for therapy. Curr Opin Nephrol Hypertens.

[45] Xu S, Han S, Dai Y, Wang L, Zhang X, Ding Y (2022). A review of the mechanism of vascular endothelial injury in immunoglobulin A vasculitis. Front Physiol.

