# Supplementary material for: Comparative proteomic analysis of glomerular proteins in IgA nephropathy and IgA vasculitis with nephritis
Source: Clin Proteomics. 2023 May 13;20:21. doi: 10.1186/s12014-023-09409-w (PMC10182656; doi:10.1186/s12014-023-09409-w)
Supplement: Supplementary file 1 — Additional file 1: table S1 Clinical characteristics of IgAN patients included in the immunohistochemical study. [file 12014_2023_9409_MOESM1_ESM.docx]

**Table S1** Clinical characteristics of IgAN patients included in the immunohistochemical study

|  | **IgAN without NS** | **IgAN with NS** |
| --- | --- | --- |
| Number of patients | 3 | 3 |
| Median age (years) (range) | 35 (19‒39) | 80 (62‒82) |
| Male:female | 1:2 | 1:2 |
| Chance proteinuria and/or hematuria, *n* | 2 | 0 |
| Gross hematuria, *n* | 1 | 0 |
| Progressive renal dysfunction, *n* | 1 | 0 |
| Edema, *n* | 0 | 3 |
| Hypertension, *n* | 0 | 2 |
| Median proteinuria (g/day or g/gCr) at biopsy (range) | 0.1 (0.1‒0.5) | 6.0 (3.7‒10.1) |
| Median serum albumin (g/dL) (range) | 4.5 (4.3‒4.6) | 2.6 (2.4‒3.0) |
| Median serum Cr (mg/dL) (range) | 0.58 (0.45‒1.15) | 0.94 (0.81‒1.08) |
| Median eGFR (mL/min/1.73 m^2^) (range) | 90.9 (60.0‒147.5) | 43.3 (40.3‒51.3) |
| Median serum IgA (mg/dL) (range) | 329 (208‒362) | 392 (243‒531) |

Cr: creatinine; eGFR: estimated glomerular filtration rate; IgAN: IgA nephropathy; NS: nephrotic syndrome
