# Supplementary material for: Comparative proteomic analysis of glomerular proteins in IgA nephropathy and IgA vasculitis with nephritis
Source: Clin Proteomics. 2023 May 13;20:21. doi: 10.1186/s12014-023-09409-w (PMC10182656; doi:10.1186/s12014-023-09409-w)
Supplement: Supplementary file 2 — Additional file 2: table S2 Pathological characteristics of IgAN patients included in the immunohistochemical study. [file 12014_2023_9409_MOESM2_ESM.docx]

**Table S2** Pathological characteristics of IgAN patients included in the immunohistochemical study

|  | **IgAN without NS** | **IgAN with NS** |
| --- | --- | --- |
| Median time from presentation to biopsy (months) (range) | 8 (4-12) | 3 (2-3) |
| Median % of global sclerotic glomeruli (range) | 11.6 (0-19.4) | 0 (0-6.3) |
| Oxford classification, *n* |  |  |
| M score | 3 M1 | 3 M1 |
| E score | 3 E0 | 1 E0, 2 E1 |
| S score | 2 S0, 1 S1 | 2 S0, 1 S1 |
| T score | 2 T0, 1 T1 | 2 T0, 1 T1 |
| C score | 3 C1 | 2 C0, 1 C1 |
| Glomerular deposition (IF intensities), *n* |  |  |
| IgA | 3 (2+) | 1 (+), 2 (3+) |
| IgG | 1 (‒), 1 (±), 1 (+) | 3 (‒) |
| IgM | 1 (‒), 2 (+) | 1 (‒), 2 (±) |
| κ | 1 (±), 2 (2+) | 2 (±), 1 (2+) |
| λ | 3 (2+) | 1 (±), 2 (2+) |
| C3 | 1 (+), 2 (2+) | 1 (‒), 2 (2+) |
| C1q | 3 (‒) | 3 (‒) |

C: cellular or fibrocellular crescents; E: endocapillary hypercellularity; IF: immunofluorescence; IgAN: IgA nephropathy; M: mesangial hypercellularity; NS: nephrotic syndrome; S: segmental glomerulosclerosis; T: tubular atrophy and interstitial fibrosis
