# Supplementary material for: Comparative proteomic analysis of glomerular proteins in IgA nephropathy and IgA vasculitis with nephritis
Source: Clin Proteomics. 2023 May 13;20:21. doi: 10.1186/s12014-023-09409-w (PMC10182656; doi:10.1186/s12014-023-09409-w)
Supplement: Supplementary file 5 — Additional file 5: table S5 List of IgA1 peptides. [file 12014_2023_9409_MOESM5_ESM.docx]

**Table S5** List of IgA1 peptides

| **NCBInr DB / GenBank DB**  **Amino acid sequence** | **Region** | **Used**  **（Not**  **shared)** | **IgAN-I (*n* = 6) /**  **Control (*n* = 5) ratio**  **(Ratio variability [%])** | **IgAN-II (*n* = 6) /**  **Control (*n* = 5) ratio**  **(Ratio variability [%])** | **IgAVN-I (*n* = 6) /**  **Control (*n* = 5) ratio**  **(Ratio variability [%])** | **IgAVN-II (*n* = 6) /**  **Control (*n* = 5) ratio**  **(Ratio variability [%])** |
| --- | --- | --- | --- | --- | --- | --- |
|  |  |  |  |  |  |  |
| 34527290 / AK130476.1 (BAC85363.1) |  |  | 100 ** | 100 ** | 100 ** | 100 ** |
| [R].QEPSQGTTTFAVTSILR.[V] #1 | C |  | 100 ** | 100 ** | 100 ** | 100 ** |
| [R].QEPSQGTTTFAVTSILR.[V] | C |  | 7.87 (22.88) | 3.59 (65.88) | 4.12 (38.08) | 4.32 (28.33) |
| [K].TPLTATLSK.[S] | C |  | 100 ** | 100 ** | 100 ** | 100 ** |
| [R].WLQGSQELPR.[E] | C |  | 16.49 ** (57.21) | 17.71 ** (94.34) | 15.88 * (72.46) | 12.91 * (60.38) |
| [K].TFTCTAAYPESK.[T] | C |  | 3.77 (136.28) | 2.20 (52.68) | 1.90 (13.04) | 3.04 (8.87) |
| [R].DASGVTFTWTPSSGK.[S] | C |  | 1.78 (116.35) | 2.39 (106.72) | 1.94 (117.70) | 1.30 (119.64) |
| [K].SAVQGPPER.[D] | C |  | 1.96 (115.26) | 3.35 (126.13) | 2.62 (128.61) | 3.82 (79.92) |
| [RK].VTVSSASPTSPK.[V] | V FR4 #2 C | Used | 100 ** | 100 ** | 100 ** | 100 ** |
| [R].VAAEDWK.[K] | C |  | 5.53 (40.43) | 3.07 (35.33) | 3.52 (42.18) | 4.84 (36.25) |
| [K].YLTWASR.[Q] | C |  | 16.39 * (43.36) | 9.90 ** (64.86) | 8.77 (48.13) | 10.63 * (71.40) |
|  |  |  |  |  |  |  |
| 34527233 / BAC85349.1 |  |  | 1.67 (106.05) | 2.02 (96.64) | 1.23 (98.81) | 1.87 (84.43) |
| [R].QEPSQGTTTFAVTSILR.[V] #1 | C |  | 100 ** | 100 ** | 100 ** | 100 ** |
| [R].QEPSQGTTTFAVTSILR.[V] | C |  | 7.87 (22.88) | 3.59 (65.88) | 4.12 (38.08) | 4.32 (28.33) |
| [K].TPLTATLSK.[S] | C |  | 100 ** | 100 ** | 100 ** | 100 ** |
| [R].WLQGSQELPR.[E] | C |  | 16.49 ** (57.21) | 17.71 ** (94.34) | 15.88 * (72.46) | 12.91 * (60.38) |
| [K].TFTCTAAYPESK.[T] | C |  | 3.77 (136.28) | 2.20 (52.68) | 1.90 (13.04) | 3.04 (8.87) |
| [R].DASGVTFTWTPSSGK.[S] | C |  | 1.78 (116.35) | 2.39 (106.72) | 1.94 (117.70) | 1.30 (119.64) |
| [R].GTLVTVSSASPTSPK.[V] | V RF4 #2 C | Used | 1.67 (106.05) | 2.02 (96.64) | 1.23 (98.81) | 1.87 (84.43) |
| [K].SAVQGPPER.[D] | C |  | 1.96 (115.26) | 3.35 (126.13) | 2.62 (128.61) | 3.82 (79.92) |
| [R].VAAEDWK.[K] | C |  | 5.53 (40.43) | 3.07 (35.33) | 3.52 (42.18) | 4.84 (36.25) |
| [K].YLTWASR.[Q] | C |  | 16.39 (43.36) | 9.90 (64.86) | 8.77 (48.13) | 10.63 (71.40) |
|  |  |  |  |  |  |  |

C: constant; DB: database; FR4: framework segment 4; IgAN: IgA nephropathy; IgAVN: IgA vasculitis with nephritis; NCBInr: National Center for Biotechnology information non-redundant; V: variable

Shared peptides in different protein entries were not used to compare protein abundance between groups.

#1 1xGln->pyro-Glu [N-Term]

#2 J Am Soc Mass Spectrom. 2021;32:1326−35.

* *P* < 0.05, ***P* < 0.01
