# Supplementary material for: Comparative proteomic analysis of glomerular proteins in IgA nephropathy and IgA vasculitis with nephritis
Source: Clin Proteomics. 2023 May 13;20:21. doi: 10.1186/s12014-023-09409-w (PMC10182656; doi:10.1186/s12014-023-09409-w)
Supplement: Supplementary file 6 — Additional file 6: table S6 List of IgG1 peptides. [file 12014_2023_9409_MOESM6_ESM.docx]

**Table S6** List of IgG1 peptides

| **NCBInr DB / GenBank DB**  **Amino acid sequence** | **Region** | **Used**  **(Not**  **shared)** | **IgAN-I (*n* = 6) /**  **Control (*n* = 5) ratio**  **(Ratio variability [%])** | **IgAN-II (*n* = 6) /**  **Control (*n* = 5) ratio**  **(Ratio variability [%])** | **IgAVN-I (*n* = 6) /**  **Control (*n* = 5) ratio**  **(Ratio variability [%])** | **IgAVN-II (*n* = 6) /**  **Control (*n* = 5) ratio**  **(Ratio variability [%])** |
| --- | --- | --- | --- | --- | --- | --- |
|  |  |  |  |  |  |  |
| 34527698 / AK130813.1 (BAC85434.1) |  |  | 100 ** | 100 ** | 100 ** | 100 ** |
| [R].TPEVTCVVVDVSHEDPEVK.[F] | C |  | 0.96 (38.39) | 2.26 (95.13) | 1.50 (72.65) | 2.87 (55.53) |
| [K].TTPPVLDSDGSFFLYSK.[LR] | C |  | 0.73 (36.83) | 2.50 (90.62) | 0.22 (12.33) | 0.90 (43.10) |
| [R].VVSVLTVLHQDWLNGK.[E] | C |  |  | 100 ** |  |  |
| [R].EPQVYTLPPSRDELTK.[N] | C |  | 3.85 (21.49) | 6.43 (37.26) | 4.77 (14.71) | 4.46 (26.22) |
| [K].GPSVFPLAPSSK.[ST-XNG] | C |  | 3.39 (61.87) | 5.48 (75.30) | 2.87 (48.44) | 4.72 (48.03) |
| [R].VTISVDTSK.[NSKIYDTARH] | V FR3 | Used | 100 ** | 100 ** | 100 ** | 100 ** |
| [K].FNWYVDGVEVHNAK.[T] | C |  |  | 100 ** |  |  |
| [R].EPQVYTLPPSR.[EDKCG] | C |  | 100 ** | 100 ** | 100 ** | 100 ** |
| [K].NQVSLTCLVK.[GA] | C |  | 1.37 (21.70) | 2.74 (47.64) | 0.52 (60.80) | 1.41 (39.62) |
|  |  |  |  |  |  |  |
| 16553682 / AK057754.1 (BAB71560.1) |  |  | 100 ** | 100 ** | 100 ** | 100 ** |
| [R].TPEVTCVVVDVSHEDPEVK.[F] | C |  | 0.96 (38.39) | 2.26 (95.13) | 1.50 (72.65) | 2.87 (55.53) |
| [K].TTPPVLDSDGSFFLYSK.[LR] | C |  | 0.73 (36.83) | 2.50 (90.62) | 0.22 (12.33) | 0.90 (43.10) |
| [R].VVSVLTVLHQDWLNGK.[E] | C |  |  | 100 ** |  |  |
| [KR].STSGGTAALGCLVK.[DA-K] # | C |  | 0.25 (52.55) | 0.68 (83.21) | 0.11 (44.85) | 0.43 (83.43) |
| [R].EPQVYTLPPSRDELTK.[N] | C |  | 3.85 (21.49) | 6.43 (37.26) | 4.77 (14.71) | 4.46 (26.22) |
| [K].GPSVFPLAPSSK.[ST-XNG] | C |  | 3.39 (61.87) | 5.48 (75.30) | 2.87 (48.44) | 4.72 (48.03) |
| [K].FNWYVDGVEVHNAK.[T] | C |  |  | 100 ** |  |  |
| [R].EPQVYTLPPSR.[EDKCG] | C |  | 100 ** | 100 ** | 100 ** | 100 ** |
| [K].NQVSLTCLVK.[GA] | C |  | 1.37 (21.70) | 2.74 (47.64) | 0.52 (60.80) | 1.41 (39.62) |
| [R].VTISADTSK.[NTADS] | V FR3 | Used | 100 ** | 100 ** | 100 ** | 100 ** |
|  |  |  |  |  |  |  |

C: constant; FR3: framework segment 3; IgAN: IgA nephropathy; IgAVN: IgA vasculitis with nephritis; NCBInr: National Center for Biotechnology information non-redundant; V: variable

Shared peptides in different protein entries were not used to compare protein abundance between groups.

# Corresponding peptide in 34527698 / AK130813.1 (BAC85434.1): STSGGTAALGCLVR

***P* < 0.01
