# Supplementary material for: Comparative proteomic analysis of glomerular proteins in IgA nephropathy and IgA vasculitis with nephritis
Source: Clin Proteomics. 2023 May 13;20:21. doi: 10.1186/s12014-023-09409-w (PMC10182656; doi:10.1186/s12014-023-09409-w)
Supplement: Supplementary file 7 — Additional file 7: table S7 List of IgM peptides. [file 12014_2023_9409_MOESM7_ESM.docx]

**Table S7** List of IgM peptides

| **NCBInr DB / PDB or GenBank DB**  **Amino acid sequence** | **Region** | **Used**  **(Not**  **shared)** | **IgAN-I (*n* = 6) /**  **Control (*n* = 5) ratio**  **(Ratio variability [%])** | **IgAN-II (*n* = 6) /**  **Control (*n* = 5) ratio**  **(Ratio variability [%])** | **IgAVN-I (*n* = 6) /**  **Control (*n* = 5) ratio**  **(Ratio variability [%])** | **IgAVN-II (*n* = 6) /**  **Control (*n* = 5) ratio**  **(Ratio variability [%])** |
| --- | --- | --- | --- | --- | --- | --- |
|  |  |  |  |  |  |  |
| 15825712 / 1HEZ_B |  |  | 100 ** | 100 ** | 100 ** | 100 ** |
| [K].YAATSQVLLPSK.[DGN-] | C |  | 100 ** | 100 ** | 100 ** | 100 ** |
| [-R].QVQLVESGGGVVQPGR.[SF] # | V FR1 | Used | 100 ** | 100 ** | 100 ** | 100 ** |
|  |  |  |  |  |  |  |
| 33451 / X17115.1 (CAA34971.1) |  |  | 5.69 (60.78) | 4.23 (45.37) | 4.72 (56.46) | 4.58 (49.63) |
| [K].YAATSQVLLPSK.[DGN-] | C |  | 100 ** | 100 ** | 100 ** | 100 ** |
| [K].YVTSAPMPEPQAPGR.[Y] | C | Used | 100 ** | 100 ** | 100 ** | 100 ** |
| [K].QVGSGVTTDQVQAEAK.[E] | C | Used | 6.73 (72.01) | 4.41 (49.48) | 5.48 (48.60) | 6.10 (37.50) |
| [R].VFAIPPSFASIFLTK.[S] | C | Used | 100 ** | 100 ** |  | 100 ** |
| [K-].VTSTLTIK.[EZ] | C | Used | 3.69 (66.21) | 3.79 (41.31) | 4.24 (62.58) | 4.05 (23.86) |
| [R].DGFFGNPR.[KS] | C | Used | 100 ** | 100 ** |  |  |
|  |  |  |  |  |  |  |

C: constant; FR1: framework segment 1; NCBInr: National Center for Biotechnology information non-redundant; PDB: Protein Data Bank; V: variable

Shared peptides in different protein entries were not used to compare protein abundance between groups.

# Corresponding peptide in 33451 / X17115.1 (CAA34971.1): QVQLVQSGAEVKKPGS

***P* < 0.01
