# Supplementary material for: Comparative proteomic analysis of glomerular proteins in IgA nephropathy and IgA vasculitis with nephritis
Source: Clin Proteomics. 2023 May 13;20:21. doi: 10.1186/s12014-023-09409-w (PMC10182656; doi:10.1186/s12014-023-09409-w)
Supplement: Supplementary file 8 — Additional file 8: table S8 List of complement C3 peptides. [file 12014_2023_9409_MOESM8_ESM.docx]

**Table S8** List of complement C3 peptides

| **Amino acid sequence** | **Residue**  **number** | **IgAN-I (*n* = 6) /**  **Control (*n* = 5) ratio**  **(Ratio variability [%])** | **IgAN-II (*n* = 6) /**  **Control (*n* = 5) ratio**  **(Ratio variability [%])** | **IgAVN-I (*n* = 6) /**  **Control (*n* = 5) ratio**  **(Ratio variability [%])** | **IgAVN-II (*n* = 6) /**  **Control (*n* = 5) ratio**  **(Ratio variability [%])** |
| --- | --- | --- | --- | --- | --- |
|  |  |  |  |  |  |
| [K].VVLVSLQSGYLFIQTDK.[T] | 120-136 |  | 100 ** | 100 ** | 100 ** |
| [K].TIYTPGSTVLYR.[I] | 137-148 | 100 ** | 100 ** | 100 ** | 100 ** |
| [K].EYVLPSFEVIVEPTEK.[F] | 226-241 | 100 ** | 100 ** | 100 ** | 100 ** |
| [K].GLEVTITAR.[F] | 250-258 | 100 ** | 100 ** | 100 ** | 100 ** |
| [R].IPIEDGSGEVVLSR.[K] | 291-304 | 100 ** | 100 ** | 100 ** | 100 ** |
| [R].LVAYYTLIGASGQR.[E] | 531-544 | 100 ** | 100 ** | 100 ** | 100 ** |
| [R].VVLVAVDK.[G] | 593-600 | 100 ** | 100 ** | 100 ** |  |
| [R].QGALELIK.[K] | 1043-1050 | 15.34 (56.32) | 12.14 * (97.64) | 6.20 (46.04) | 4.55 (72.11) |
| [K].KGYTQQLAFR.[Q] | 1051-1060 | 100 ** | 100 ** | 100 ** | 100 ** |
| [K].GYTQQLAFR.[Q] | 1052-1060 | 23.28 ** (52.91) | 12.34 ** (113.35) | 11.35 * (57.88) | 5.31 (77.86) |
| [R].QPSSAFAAFVK.[R] | 1061-1071 | 13.59 (58.71) | 22.28 ** (62.00) | 10.05 (70.23) | 7.29 (130.77) |
| [K].DICEEQVNSLPGSITK.[A] | 1156-1171 | 100 ** | 100 ** | 100 ** | 100 ** |
| [K].AGDFLEANYMNLQR.[S] | 1172-1185 | 100 ** |  | 100 ** | 100 ** |
| [R].LKGPLLNK.[F] | 1202-1209 | 100 ** | 100 ** | 100 ** | 100 ** |
| [K].DFDFVPPVVR.[W] | 1245-1254 | 8.92 (68.05) | 8.43 * (107.64) | 6.37 (42.92) | 2.14 (12.38) |
| [K].SGSDEVQVGQQR.[T] | 1571-1582 | 100 ** | 100 ** | 100 ** | 100 ** |
|  |  |  |  |  |  |

IgAN: IgA nephropathy; IgAVN: IgA vasculitis with nephritis

* *P* < 0.05, ***P* < 0.01
