# Supplementary figures and images for: Comparative proteomic analysis of glomerular proteins in IgA nephropathy and IgA vasculitis with nephritis
Source: Clin Proteomics. 2023 May 13;20:21. doi: 10.1186/s12014-023-09409-w (PMC10182656; doi:10.1186/s12014-023-09409-w)

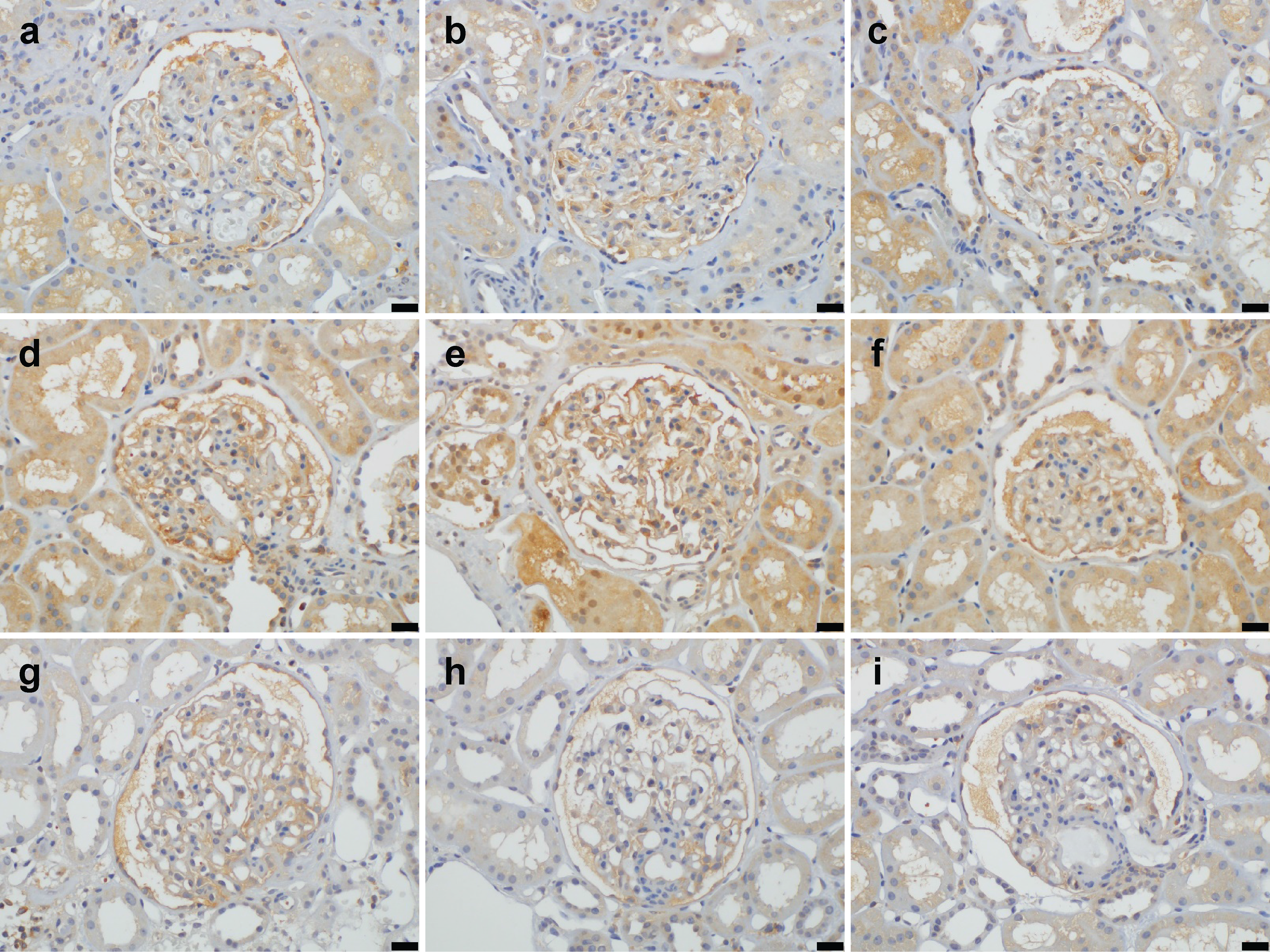

Supplement: Supplementary file 9 — Additional file 9: figure S1 Immunohistochemical staining for talin 1 in IgAN patients without NS. a-c: A 35-year-old male; d-f: a 19-year-old female; g-i: a 39-year-old female. Bars represent 20 μm. Original magnification of each image: ×400. [file 12014_2023_9409_MOESM9_ESM.tif]

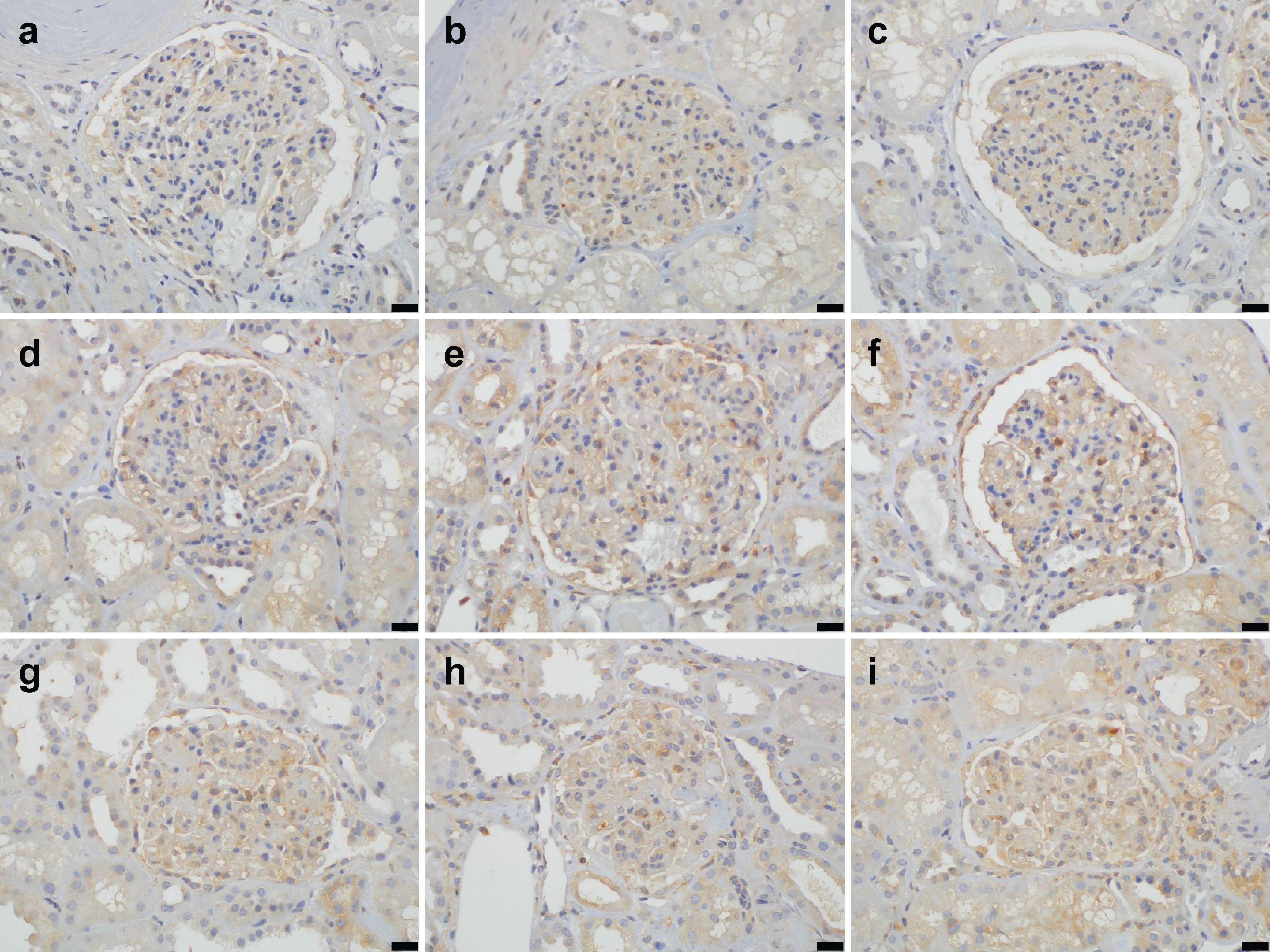

Supplement: Supplementary file 10 — Additional file 10: figure S2 Immunohistochemical staining for talin 1 in IgAN patients with NS. a-c: An 80-year-old female; d-f: a 62-year-old male; g-i: a 82-year-old female. Bars represent 20 μm. Original magnification of each image: ×400. [file 12014_2023_9409_MOESM10_ESM.tif]
